# *cyclo*-Tetra-μ-oxido-tetra­kis­[(acetyl­acetonato-κ^2^ *O*,*O*′)bis­(ethano­lato-κ*O*)niobium(V)]

**DOI:** 10.1107/S1600536811044138

**Published:** 2011-11-05

**Authors:** Leandra Herbst, Hendrik G. Visser, Andreas Roodt, Theunis J. Muller

**Affiliations:** a Department of Chemistry, University of the Free State, 9300 Bloemfontein, South Africa

## Abstract

The asymmetric unit of the title tetra­nuclear niobium(V) compound, [Nb_4_(C_2_H_5_O)_8_(C_5_H_7_O_2_)_4_O_4_], contains two Nb^V^ atoms, two bridging O atoms, two acetyl­acetonate and four ethano­late ligands. Each Nb^V^ atom is six-coordinated by the bridging O atoms, two ethano­late and one chelating acetyl­acetonate ligands. The Nb—O distances vary between 1.817 (2) and 2.201 (2) Å and the O—Nb—O angles vary between 78.88 (8) and 102.78 (9)°, illustrating the significant distortion from ideal ocahedral geometry. The rest of the tetra­nuclear unit is generated through an inversion centre. The C atoms of two of the ethano­late mol­ecules are disordered over two sites [occupancy ratio 0.601 (12):0.399 (12)].

## Related literature

For similar structures, see: Ooi & Sotofte (2004 ▶); Cotton *et al.* (1985 ▶, 1987 ▶); Steunou *et al.* (1998 ▶). For applications of acetyl­acetone in industry, see: Steyn *et al.* (1992 ▶, 1997 ▶, 2008 ▶); Otto *et al.* (1998 ▶); Roodt & Steyn (2000 ▶); Brink *et al.* (2010 ▶); Viljoen *et al.* (2008 ▶, 2009**a* ▶,b* ▶, 2010 ▶); Herbst *et al.* (2010 ▶). For a review article about structure–reactivity relationships, see: Roodt *et al.* (2011 ▶)
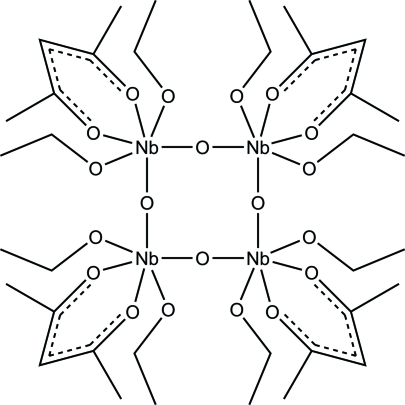


## Experimental

### 

#### Crystal data

[Nb_4_(C_2_H_5_O)_8_(C_5_H_7_O_2_)_4_O_4_]
*M* *_r_* = 1192.54
Monoclinic, 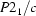

*a* = 13.907 (5) Å
*b* = 12.662 (5) Å
*c* = 21.354 (5) Å
β = 136.982 (13)°
*V* = 2565.4 (15) Å^3^
*Z* = 2
Mo *K*α radiation
μ = 0.94 mm^−1^
*T* = 180 K
0.48 × 0.32 × 0.27 mm

#### Data collection

Bruker X8 APEXII 4K Kappa CCD diffractometer
Absorption correction: multi-scan (*SADABS*; Bruker, 2004 ▶) *T*_min_ = 0.701, *T*_max_ = 0.778
42149 measured reflections
6191 independent reflections
5355 reflections with *I* > 2σ(*I*)
*R*_int_ = 0.032

#### Refinement

*R*[*F* ^2^ > 2σ(*F* ^2^)] = 0.031
*wR*(*F* ^2^) = 0.080
*S* = 1.06
6191 reflections
310 parameters
85 restraints
H-atom parameters constrained
Δρ_max_ = 2.43 e Å^−3^
Δρ_min_ = −1.32 e Å^−3^

### 

Data collection: *APEX2* (Bruker, 2010 ▶); cell refinement: *SAINT-Plus* (Bruker, 2004 ▶); data reduction: *SAINT-Plus*; program(s) used to solve structure: *SHELXS97* (Sheldrick, 2008 ▶); program(s) used to refine structure: *SHELXL97* (Sheldrick, 2008 ▶); molecular graphics: *DIAMOND* (Brandenburg & Putz, 2005 ▶); software used to prepare material for publication: *WinGX* (Farrugia, 1999 ▶).

## Supplementary Material

Crystal structure: contains datablock(s) global, I. DOI: 10.1107/S1600536811044138/bg2426sup1.cif

Structure factors: contains datablock(s) I. DOI: 10.1107/S1600536811044138/bg2426Isup2.hkl

Additional supplementary materials:  crystallographic information; 3D view; checkCIF report

## Figures and Tables

**Fig. 1.**
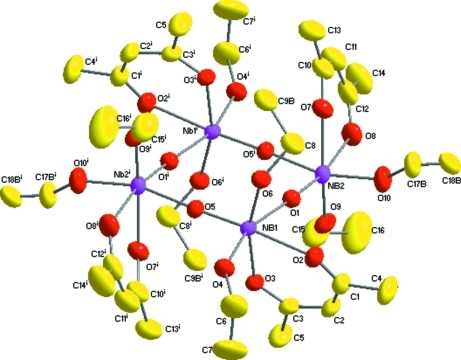
Molecular structure of the title compound. Displacement ellipsoids are drawn at the 50% probability level. Hydrogen atoms ommitted for clarity. Symmetry code: (i) -x + 2, -y + 1, -z + 1.

**Fig. 2.**
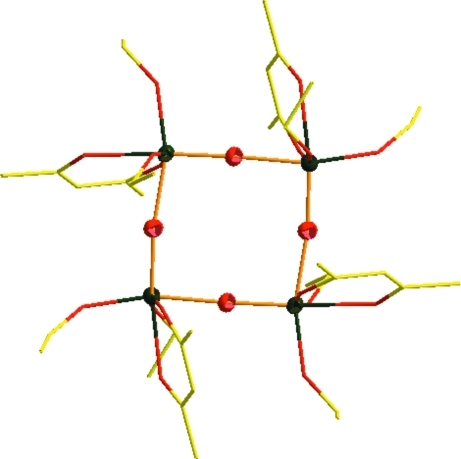
Square Nb–O arrangement in the molecule.

## supplementary crystallographic information

### Comment

Acetylacetone finds applications in homgenous catalysis and the separations industry (Steyn *et al.*, 1992, 1997; Otto *et al.*, 1998; Roodt & Steyn, 2000; Brink *et al.*, 2010). This study forms part of ongoing research to investigate the intimate mechanism of the reactions of polidentate ligands with transition metals used in the nuclear industry, especially hafnium, zirconium, niobium and tantalum (Viljoen *et al.*, 2008,2009*a*,2009*b*, 2010; Steyn *et al.*, 2008; Herbst *et al.*, 2010; Roodt *et al.*, 2011).

In the title tetranuclear Niobium(V) compound, [Nb(CH_3_CH_2_O)_2_(C_5_H_7_O_2_)(µ^2^-O)]_4_, the asymmetric unit contains two niobium atoms, separated by a bridging oxygen atom, two acetylacetonato bidentate ligands, four ethanolate ligands and another bridging oxygen atom coordinated to Nb1. The rest of the title compound is generated through an inversion centre (see Figure 1).

Each niobium atom is six coordinated to two bridging oxygen atoms, two ethanolate molecules and a chelating acetylacetonato ligand. The Nb–O distances vary between 1.817 (2) to 2.201 (3) Å and the O–Nb–O angles vary between 78.86 (10) and 102.79 (11) °, illustrating the significant distortion from ideal octahedral geometry. The most significant deviation from the ideal 180 ° of the *trans* O–Nb–O angles is obtained for O6–Nb1– O3, namely 163.66 (10) °. All the bond distances and angles are similar to relevant niobium(V) structures (Ooi *et al.*, 2004; Cotton *et al.*, 1985, 1987; Steunou *et al.*, 1998).

The four niobium atoms and the four bridging oxygen atoms form a slightly distorted square with Nb–Nb distances of 3.8339 (13) and 3.8229 (9) ° respectively and O–Nb–O angles of 93.526 (14) and 97.123 (13) Å (see Figure 2). The planarity of this square arrangement is indicated by the small distances that the Nb and O atoms are protruding from a plane generated through Nb1, Nb2, O1 and O5; the largest distance from the plane being 0.575 (14) Å, obtained for O1.

Two of the carbon atoms of one of the ethanolate ligands are disordered over two positions (53% to 47%) while the methyl carbon of another ethanolate ligand displays a vibrational disorder of 72%. Two of the ethanolate molecules are disordered over two positions.

### Experimental

The reaction was performed under modified Schlenk conditions under an argon atmosphere. Nb(OEt)_5_ (1.16 mmol, 0.291 ml) and acetylacetone (1.16 mmol, 0.119 ml) were added together and stirred for 30 min. Absolute methanol (5 ml) was added to the reaction mixture and allowed to stir for another 30 min at room temperature. The colourless solution was left to stand at 252 K for a few days after which white crystals, suitable for X-ray diffraction were obtained.

### Refinement

The methine and methylene H atoms were placed in geometrically idealized positions at C—H = 0.93 and 0.97 Å, respectively and constrained to ride on their parent atoms, with *U*_iso_(H) = 1.2*U*_eq_(C). The highest peak is located 0.81 Å from NB2 and the deepest hole is situated 0.67 Å from Nb2.

A larger than usual U(eq) range for the disordered methyl atoms is observed and were refined using the DELU and SIMU instructions.

A few reflections were influenced by the beamstop and therefore omitted to obtain a better refinement.

### Crystal data

**Table:**

[Nb_4_(C_2_H_5_O)_8_(C_5_H_7_O_2_)_4_O_4_]	*F*(000) = 1216
*M**_r_* = 1192.54	*D*_x_ = 1.544 Mg m^−^^3^
Monoclinic, *P*2_1_/*c*	Mo *K*α radiation, λ = 0.71073 Å
Hall symbol: -P 2ybc	Cell parameters from 9935 reflections
*a* = 13.907 (5) Å	θ = 2.7–28.3°
*b* = 12.662 (5) Å	µ = 0.94 mm^−^^1^
*c* = 21.354 (5) Å	*T* = 180 K
β = 136.982 (13)°	Cuboid, colourless
*V* = 2565.4 (15) Å^3^	0.48 × 0.32 × 0.27 mm
*Z* = 2	

### Data collection

**Table:**

Bruker X8 APEXII 4K Kappa CCD diffractometer	6191 independent reflections
Radiation source: fine-focus sealed tube	5355 reflections with *I* > 2σ(*I*)
graphite	*R*_int_ = 0.032
φ and ω scans	θ_max_ = 28°, θ_min_ = 3.2°
Absorption correction: multi-scan (*SADABS*; Bruker, 2004)	*h* = −18→18
*T*_min_ = 0.701, *T*_max_ = 0.778	*k* = −16→16
42149 measured reflections	*l* = −25→28

### Refinement

**Table:**

Refinement on *F*^2^	Primary atom site location: structure-invariant direct methods
Least-squares matrix: full	Secondary atom site location: difference Fourier map
*R*[*F*^2^ > 2σ(*F*^2^)] = 0.031	Hydrogen site location: inferred from neighbouring sites
*wR*(*F*^2^) = 0.080	H-atom parameters constrained
*S* = 1.06	*w* = 1/[σ^2^(*F*_o_^2^) + (0.031*P*)^2^ + 4.1146*P*] where *P* = (*F*_o_^2^ + 2*F*_c_^2^)/3
6191 reflections	(Δ/σ)_max_ = 0.002
310 parameters	Δρ_max_ = 2.43 e Å^−^^3^
85 restraints	Δρ_min_ = −1.32 e Å^−^^3^

### Special details

**Table:**

Experimental. The intensity data were collected on a Bruker X8 ApexII 4 K Kappa CCD diffractometer using an exposure time of 40 s/frame. A total of 1709 frames were collected with a frame width of 0.5° covering up to θ = 28.39° with 99.9% completeness accomplished.
Geometry. All s.u.'s (except the s.u. in the dihedral angle between two l.s. planes) are estimated using the full covariance matrix. The cell s.u.'s are taken into account individually in the estimation of s.u.'s in distances, angles and torsion angles; correlations between s.u.'s in cell parameters are only used when they are defined by crystal symmetry. An approximate (isotropic) treatment of cell s.u.'s is used for estimating s.u.'s involving l.s. planes.
Refinement. Refinement of *F*^2^ against ALL reflections. The weighted *R*-factor *wR* and goodness of fit *S* are based on *F*^2^, conventional *R*-factors *R* are based on *F*, with *F* set to zero for negative *F*^2^. The threshold expression of *F*^2^ > 2σ(*F*^2^) is used only for calculating *R*-factors(gt) *etc*. and is not relevant to the choice of reflections for refinement. *R*-factors based on *F*^2^ are statistically about twice as large as those based on *F*, and *R*- factors based on ALL data will be even larger.

### Fractional atomic coordinates and isotropic or equivalent isotropic displacement parameters (Å^2^)

**Table:**

	*x*	*y*	*z*	*U*_iso_*/*U*_eq_	Occ. (<1)
C1	0.4871 (3)	0.5567 (3)	0.2203 (2)	0.0376 (6)	
C2	0.5266 (3)	0.6468 (3)	0.2724 (2)	0.0440 (7)	
H2	0.4807	0.7098	0.2414	0.053*	
C3	0.6286 (3)	0.6484 (2)	0.3664 (2)	0.0377 (6)	
C4	0.3652 (4)	0.5634 (3)	0.1170 (2)	0.0635 (11)	
H4A	0.3966	0.544	0.0908	0.095*	
H4B	0.3295	0.6344	0.0993	0.095*	
H4C	0.2918	0.5161	0.0952	0.095*	
C5	0.6564 (5)	0.7475 (3)	0.4162 (3)	0.0597 (10)	
H5A	0.6275	0.7376	0.445	0.09*	
H5B	0.6043	0.8049	0.3732	0.09*	
H5C	0.7553	0.7635	0.462	0.09*	
C6	0.4762 (4)	0.3502 (4)	0.3446 (3)	0.0611 (10)	
H6A	0.4608	0.283	0.3581	0.073*	
H6B	0.4148	0.3529	0.2791	0.073*	
C7	0.4382 (5)	0.4360 (5)	0.3696 (4)	0.0893 (17)	
H7A	0.3415	0.4284	0.336	0.134*	
H7B	0.4506	0.5027	0.3548	0.134*	
H7C	0.4976	0.4331	0.4342	0.134*	
C8	0.7632 (4)	0.2881 (3)	0.2822 (3)	0.0485 (8)	
H8A	0.8199	0.3423	0.2892	0.058*	
H8B	0.6702	0.2895	0.2191	0.058*	
C10	1.0999 (3)	0.4854 (3)	0.3539 (2)	0.0402 (7)	
C11	1.1829 (4)	0.5716 (3)	0.3770 (3)	0.0494 (8)	
H11	1.2651	0.5589	0.3937	0.059*	
C12	1.1506 (4)	0.6763 (3)	0.3769 (2)	0.0468 (8)	
C13	1.1348 (4)	0.3772 (3)	0.3489 (3)	0.0571 (9)	
H13A	1.0784	0.36	0.2861	0.086*	
H13B	1.116	0.3276	0.3729	0.086*	
H13C	1.2328	0.3739	0.3844	0.086*	
C14	1.2474 (5)	0.7644 (4)	0.4018 (3)	0.0672 (12)	
H14A	1.3141	0.7374	0.4039	0.101*	
H14B	1.2967	0.7925	0.4609	0.101*	
H14C	1.1926	0.8194	0.3566	0.101*	
C15	0.9542 (5)	0.8296 (3)	0.4372 (3)	0.0672 (11)	
H15A	1.0526	0.81	0.4879	0.081*	
H15B	0.921	0.8503	0.4625	0.081*	
C16	0.9434 (7)	0.9182 (4)	0.3908 (4)	0.103 (2)	
H16A	0.8466	0.9396	0.3416	0.155*	
H16B	0.9777	0.8989	0.3665	0.155*	
H16C	0.9986	0.9755	0.4333	0.155*	
O1	0.81776 (19)	0.51714 (15)	0.36184 (13)	0.0296 (4)	
O2	0.54526 (19)	0.46715 (16)	0.25306 (13)	0.0342 (4)	
O3	0.7048 (2)	0.56870 (15)	0.41852 (13)	0.0323 (4)	
O4	0.6178 (2)	0.35591 (17)	0.39197 (14)	0.0351 (4)	
O5	0.90700 (18)	0.40537 (14)	0.51497 (12)	0.0290 (4)	
O6	0.7517 (2)	0.31023 (15)	0.34129 (13)	0.0322 (4)	
O7	0.9881 (2)	0.49253 (17)	0.33447 (14)	0.0362 (4)	
O8	1.0433 (2)	0.70416 (18)	0.35616 (16)	0.0434 (5)	
O9	0.8765 (2)	0.74043 (15)	0.38001 (14)	0.0386 (5)	
O10	0.7637 (2)	0.62579 (19)	0.22326 (14)	0.0458 (5)	
Nb1	0.74205 (2)	0.422519 (18)	0.394088 (15)	0.02353 (7)	
Nb2	0.90559 (2)	0.611158 (19)	0.352545 (16)	0.02815 (7)	
C17A	0.7186 (18)	0.6351 (11)	0.1421 (9)	0.075 (4)	0.399 (12)
H17A	0.7289	0.7091	0.1364	0.09*	0.399 (12)
H17B	0.618	0.6213	0.0954	0.09*	0.399 (12)
C18A	0.7686 (18)	0.5806 (10)	0.1159 (9)	0.088 (5)	0.399 (12)
H18A	0.6919	0.5624	0.0521	0.132*	0.399 (12)
H18B	0.8142	0.5173	0.1519	0.132*	0.399 (12)
H18C	0.8352	0.6235	0.1253	0.132*	0.399 (12)
C17B	0.7207 (17)	0.5822 (11)	0.1469 (7)	0.145 (7)	0.601 (12)
H17C	0.7969	0.5989	0.1545	0.174*	0.601 (12)
H17D	0.7274	0.5067	0.1572	0.174*	0.601 (12)
C18B	0.6150 (15)	0.5917 (14)	0.0646 (7)	0.184 (8)	0.601 (12)
H18D	0.5957	0.6654	0.049	0.276*	0.601 (12)
H18E	0.5366	0.5595	0.0484	0.276*	0.601 (12)
H18F	0.6292	0.5578	0.0317	0.276*	0.601 (12)
C9A	0.828 (3)	0.1850 (12)	0.3022 (14)	0.068 (4)	0.53 (5)
H9A1	0.9206	0.1841	0.3642	0.102*	0.53 (5)
H9A2	0.8335	0.1718	0.2608	0.102*	0.53 (5)
H9A3	0.7713	0.1312	0.2948	0.102*	0.53 (5)
C9B	0.885 (4)	0.218 (3)	0.330 (2)	0.076 (7)	0.47 (5)
H9B1	0.9691	0.2514	0.3856	0.114*	0.47 (5)
H9B2	0.8959	0.2052	0.2909	0.114*	0.47 (5)
H9B3	0.8706	0.1524	0.3442	0.114*	0.47 (5)

### Atomic displacement parameters (Å^2^)

**Table:**

	*U*^11^	*U*^22^	*U*^33^	*U*^12^	*U*^13^	*U*^23^
C1	0.0264 (13)	0.0447 (17)	0.0305 (14)	0.0065 (12)	0.0172 (12)	0.0082 (12)
C2	0.0424 (17)	0.0394 (16)	0.0411 (17)	0.0187 (13)	0.0276 (15)	0.0129 (13)
C3	0.0385 (15)	0.0342 (15)	0.0430 (17)	0.0098 (12)	0.0306 (14)	0.0032 (12)
C4	0.050 (2)	0.067 (3)	0.0319 (17)	0.0135 (19)	0.0168 (17)	0.0112 (16)
C5	0.073 (3)	0.0414 (19)	0.055 (2)	0.0217 (18)	0.044 (2)	0.0049 (16)
C6	0.0350 (17)	0.085 (3)	0.065 (2)	−0.0114 (18)	0.0373 (19)	−0.001 (2)
C7	0.051 (2)	0.138 (5)	0.096 (4)	0.005 (3)	0.059 (3)	−0.010 (3)
C8	0.061 (2)	0.0451 (18)	0.053 (2)	−0.0026 (15)	0.0460 (19)	−0.0075 (15)
C10	0.0412 (16)	0.0536 (19)	0.0353 (15)	−0.0021 (14)	0.0310 (14)	−0.0027 (13)
C11	0.0457 (18)	0.065 (2)	0.053 (2)	−0.0088 (16)	0.0408 (18)	−0.0069 (17)
C12	0.0495 (19)	0.058 (2)	0.0404 (17)	−0.0157 (16)	0.0354 (16)	−0.0031 (15)
C13	0.063 (2)	0.058 (2)	0.069 (3)	0.0047 (18)	0.054 (2)	−0.0039 (19)
C14	0.072 (3)	0.077 (3)	0.068 (3)	−0.037 (2)	0.056 (2)	−0.017 (2)
C15	0.069 (3)	0.043 (2)	0.061 (2)	−0.0029 (18)	0.038 (2)	−0.0100 (18)
C16	0.124 (5)	0.041 (2)	0.084 (4)	−0.015 (3)	0.057 (4)	−0.007 (2)
O1	0.0278 (9)	0.0305 (9)	0.0309 (10)	−0.0011 (7)	0.0216 (8)	0.0001 (8)
O2	0.0233 (9)	0.0367 (11)	0.0271 (9)	0.0011 (8)	0.0134 (8)	0.0016 (8)
O3	0.0328 (10)	0.0305 (10)	0.0325 (10)	0.0072 (8)	0.0235 (9)	0.0019 (8)
O4	0.0279 (9)	0.0416 (11)	0.0370 (11)	−0.0051 (8)	0.0241 (9)	−0.0019 (9)
O5	0.0244 (9)	0.0302 (9)	0.0264 (9)	−0.0001 (7)	0.0167 (8)	0.0008 (7)
O6	0.0343 (10)	0.0286 (10)	0.0345 (10)	−0.0007 (8)	0.0254 (9)	−0.0033 (8)
O7	0.0365 (10)	0.0408 (11)	0.0386 (11)	−0.0071 (9)	0.0298 (10)	−0.0068 (9)
O8	0.0483 (13)	0.0421 (12)	0.0479 (13)	−0.0052 (10)	0.0378 (12)	0.0059 (10)
O9	0.0347 (10)	0.0269 (10)	0.0412 (11)	0.0046 (8)	0.0236 (10)	0.0015 (8)
O10	0.0421 (12)	0.0540 (14)	0.0261 (10)	0.0004 (10)	0.0201 (10)	0.0054 (9)
Nb1	0.01917 (11)	0.02425 (12)	0.02382 (12)	0.00004 (8)	0.01465 (10)	−0.00014 (8)
Nb2	0.02496 (12)	0.03010 (13)	0.02436 (12)	−0.00099 (9)	0.01643 (11)	0.00325 (9)
C17A	0.111 (9)	0.059 (8)	0.047 (6)	0.011 (7)	0.056 (6)	0.018 (6)
C18A	0.149 (14)	0.080 (9)	0.066 (8)	−0.016 (8)	0.088 (10)	−0.010 (6)
C17B	0.209 (12)	0.087 (8)	0.041 (4)	0.084 (8)	0.060 (6)	0.026 (5)
C18B	0.122 (11)	0.31 (2)	0.062 (5)	0.006 (11)	0.048 (6)	−0.063 (9)
C9A	0.113 (12)	0.048 (6)	0.084 (8)	0.008 (6)	0.085 (9)	−0.009 (5)
C9B	0.107 (14)	0.075 (13)	0.098 (13)	0.051 (10)	0.091 (12)	0.045 (9)

### Geometric parameters (Å, °)

**Table:**

C1—O2	1.266 (4)	C15—C16	1.429 (6)
C1—C2	1.396 (5)	C15—H15A	0.97
C1—C4	1.509 (4)	C15—H15B	0.97
C2—C3	1.370 (4)	C16—H16A	0.96
C2—H2	0.93	C16—H16B	0.96
C3—O3	1.286 (3)	C16—H16C	0.96
C3—C5	1.501 (4)	O1—Nb2	1.8173 (19)
C4—H4A	0.96	O1—Nb1	2.0196 (19)
C4—H4B	0.96	O2—Nb1	2.197 (2)
C4—H4C	0.96	O3—Nb1	2.089 (2)
C5—H5A	0.96	O4—Nb1	1.894 (2)
C5—H5B	0.96	O5—Nb1	1.8204 (19)
C5—H5C	0.96	O5—Nb2^i^	2.0145 (19)
C6—O4	1.412 (4)	O6—Nb1	1.8793 (19)
C6—C7	1.468 (6)	O7—Nb2	2.090 (2)
C6—H6A	0.97	O8—Nb2	2.201 (2)
C6—H6B	0.97	O9—Nb2	1.880 (2)
C7—H7A	0.96	O10—C17A	1.349 (12)
C7—H7B	0.96	O10—C17B	1.377 (11)
C7—H7C	0.96	O10—Nb2	1.893 (2)
C8—O6	1.411 (4)	Nb2—O5^i^	2.0145 (19)
C8—C9A	1.460 (15)	C17A—C18A	1.357 (15)
C8—C9B	1.476 (16)	C17A—H17A	0.97
C8—H8A	0.97	C17A—H17B	0.97
C8—H8B	0.97	C18A—H18A	0.96
C10—O7	1.287 (4)	C18A—H18B	0.96
C10—C11	1.390 (5)	C18A—H18C	0.96
C10—C13	1.484 (5)	C17B—C18B	1.219 (12)
C11—C12	1.398 (5)	C17B—H17C	0.97
C11—H11	0.93	C17B—H17D	0.97
C12—O8	1.258 (4)	C18B—H18D	0.96
C12—C14	1.514 (5)	C18B—H18E	0.96
C13—H13A	0.96	C18B—H18F	0.96
C13—H13B	0.96	C9A—H9A1	0.96
C13—H13C	0.96	C9A—H9A2	0.96
C14—H14A	0.96	C9A—H9A3	0.96
C14—H14B	0.96	C9B—H9B1	0.96
C14—H14C	0.96	C9B—H9B2	0.96
C15—O9	1.415 (4)	C9B—H9B3	0.96
			
O2—C1—C2	124.8 (3)	C15—C16—H16B	109.5
O2—C1—C4	116.1 (3)	H16A—C16—H16B	109.5
C2—C1—C4	119.1 (3)	C15—C16—H16C	109.5
C3—C2—C1	124.3 (3)	H16A—C16—H16C	109.5
C3—C2—H2	117.9	H16B—C16—H16C	109.5
C1—C2—H2	117.9	Nb2—O1—Nb1	170.20 (11)
O3—C3—C2	124.9 (3)	C1—O2—Nb1	129.84 (19)
O3—C3—C5	114.9 (3)	C3—O3—Nb1	132.58 (19)
C2—C3—C5	120.3 (3)	C6—O4—Nb1	144.2 (2)
C1—C4—H4A	109.5	Nb1—O5—Nb2^i^	177.29 (11)
C1—C4—H4B	109.5	C8—O6—Nb1	142.3 (2)
H4A—C4—H4B	109.5	C10—O7—Nb2	133.4 (2)
C1—C4—H4C	109.5	C12—O8—Nb2	129.8 (2)
H4A—C4—H4C	109.5	C15—O9—Nb2	139.6 (2)
H4B—C4—H4C	109.5	C17A—O10—Nb2	153.1 (7)
C3—C5—H5A	109.5	C17B—O10—Nb2	140.8 (5)
C3—C5—H5B	109.5	O5—Nb1—O6	101.06 (9)
H5A—C5—H5B	109.5	O5—Nb1—O4	99.35 (9)
C3—C5—H5C	109.5	O6—Nb1—O4	96.49 (9)
H5A—C5—H5C	109.5	O5—Nb1—O1	97.11 (8)
H5B—C5—H5C	109.5	O6—Nb1—O1	87.74 (8)
O4—C6—C7	112.7 (3)	O4—Nb1—O1	161.86 (8)
O4—C6—H6A	109.1	O5—Nb1—O3	92.12 (8)
C7—C6—H6A	109.1	O6—Nb1—O3	163.72 (8)
O4—C6—H6B	109.1	O4—Nb1—O3	90.68 (9)
C7—C6—H6B	109.1	O1—Nb1—O3	81.13 (8)
H6A—C6—H6B	107.8	O5—Nb1—O2	171.80 (8)
C6—C7—H7A	109.5	O6—Nb1—O2	86.33 (8)
C6—C7—H7B	109.5	O4—Nb1—O2	83.19 (9)
H7A—C7—H7B	109.5	O1—Nb1—O2	79.48 (8)
C6—C7—H7C	109.5	O3—Nb1—O2	80.01 (8)
H7A—C7—H7C	109.5	O1—Nb2—O9	102.78 (9)
H7B—C7—H7C	109.5	O1—Nb2—O10	98.99 (10)
O6—C8—C9A	111.3 (7)	O9—Nb2—O10	97.89 (10)
O6—C8—C9B	109.2 (9)	O1—Nb2—O5^i^	93.52 (8)
O6—C8—H8A	109.4	O9—Nb2—O5^i^	90.28 (8)
C9A—C8—H8A	109.4	O10—Nb2—O5^i^	163.13 (9)
C9B—C8—H8A	85.6	O1—Nb2—O7	93.03 (8)
O6—C8—H8B	109.4	O9—Nb2—O7	162.89 (9)
C9A—C8—H8B	109.4	O10—Nb2—O7	85.90 (10)
C9B—C8—H8B	131.4	O5^i^—Nb2—O7	82.15 (8)
H8A—C8—H8B	108	O1—Nb2—O8	169.64 (8)
O7—C10—C11	123.6 (3)	O9—Nb2—O8	84.44 (9)
O7—C10—C13	115.1 (3)	O10—Nb2—O8	87.20 (10)
C11—C10—C13	121.4 (3)	O5^i^—Nb2—O8	78.88 (8)
C10—C11—C12	124.2 (3)	O7—Nb2—O8	79.06 (9)
C10—C11—H11	117.9	O10—C17A—C18A	126.0 (13)
C12—C11—H11	117.9	O10—C17A—H17A	105.8
O8—C12—C11	124.2 (3)	C18A—C17A—H17A	105.8
O8—C12—C14	115.9 (4)	O10—C17A—H17B	105.8
C11—C12—C14	119.8 (3)	C18A—C17A—H17B	105.8
C10—C13—H13A	109.5	H17A—C17A—H17B	106.2
C10—C13—H13B	109.5	C18B—C17B—O10	133.5 (13)
H13A—C13—H13B	109.5	C18B—C17B—H17C	103.8
C10—C13—H13C	109.5	O10—C17B—H17C	103.8
H13A—C13—H13C	109.5	C18B—C17B—H17D	103.8
H13B—C13—H13C	109.5	O10—C17B—H17D	103.8
C12—C14—H14A	109.5	H17C—C17B—H17D	105.4
C12—C14—H14B	109.5	C17B—C18B—H18D	109.5
H14A—C14—H14B	109.5	C17B—C18B—H18E	109.5
C12—C14—H14C	109.5	H18D—C18B—H18E	109.5
H14A—C14—H14C	109.5	C17B—C18B—H18F	109.5
H14B—C14—H14C	109.5	H18D—C18B—H18F	109.5
O9—C15—C16	113.7 (4)	H18E—C18B—H18F	109.5
O9—C15—H15A	108.8	C8—C9A—H9A1	109.5
C16—C15—H15A	108.8	C8—C9A—H9A2	109.5
O9—C15—H15B	108.8	C8—C9A—H9A3	109.5
C16—C15—H15B	108.8	C8—C9B—H9B1	109.5
H15A—C15—H15B	107.7	C8—C9B—H9B2	109.5
C15—C16—H16A	109.5	C8—C9B—H9B3	109.5

Symmetry codes: (i) −*x*+2, −*y*+1, −*z*+1.
